# Exploring Breastfeeding Practices and Obstacles Among Mothers With and Without Congenital Heart Disease

**DOI:** 10.3390/healthcare13243284

**Published:** 2025-12-15

**Authors:** Fahad Abdulaziz Alrashed, Saad M. AlAngari, Abdullah Awartani, Saud Alshalan, Sultan Alabdullah, Faisal Subait, Majd Alkhateeb, Sarah Ismail Mazi, Ahmed Othman Alsabih, Zafar Iqbal, Tauseef Ahmad

**Affiliations:** 1 Department of Medical Education, College of Medicine, King Saud University, P.O. Box 2925, Riyadh 11472, Saudi Arabia; tahmad@ksu.edu.sa; 2 College of Medicine, King Saud University, Riyadh 11472, Saudi Arabia; saadalangari0@gmail.com (S.M.A.); awartaniabdullah@gmail.com (A.A.); saudalshalan1@gmail.com (S.A.); sultanalabdullah98@gmail.com (S.A.); faisalalsubait17@gmail.com (F.S.); majd.nader1234@gmail.com (M.A.); 3 Department of Cardiac Sciences, College of Medicine, King Saud University, P.O. Box 7805, Riyadh 11472, Saudi Arabia; smazi@ksu.edu.sa; 4 Department of Physiology, College of Medicine, King Saud University, Riyadh 11461, Saudi Arabia; aalsabih@ksu.edu.sa; 5 Department of General Surgery, College of Medicine, King Saud University, P.O. Box 7805, Riyadh 11472, Saudi Arabia; zsyed@ksu.edu.sa

**Keywords:** congenital heart disease, breastfeeding practices, mothers with CHD, child CHD, breastfeeding obstacles, medical education, health-care education

## Abstract

**Background:** Breastfeeding provides optimal nutrition and immunological protection, offering critical benefits for infants with congenital heart disease (CHD) and their mothers. This study aims to determine the prevalence of breastfeeding practices and identify common obstacles among mothers with and without CHD whose children are diagnosed with CHD. **Methods:** This cross-sectional study assessed breastfeeding prevalence and obstacles among mothers with and without congenital heart disease (CHD) whose children were also diagnosed with CHD. Data were collected from January to August 2025 across tertiary hospitals and pediatric cardiology units in Saudi Arabia. A validated bilingual questionnaire (Cronbach’s α = 0.816) captured maternal, infant, and breastfeeding-related factors. Descriptive statistics, chi-square tests, and odds ratios were applied, with significance set at *p* < 0.05. **Results:** A total of 419 mothers of children with congenital heart disease (CHD) participated. Maternal CHD was reported in 15.8% of participants and was significantly associated with older maternal age, family history of CHD, low birth weight, and the presence of other chronic diseases (*p* < 0.05). Ventricular septal defect (VSD) and mitral stenosis were more frequent among children of CHD mothers. Breastfeeding initiation (≈91%) and duration did not differ significantly between CHD and non-CHD mothers. Interestingly, CHD mothers reported fewer breastfeeding obstacles (27.3% vs. 43.1%, *p* = 0.04) and were less likely to cite fatigue or pain as reasons for discontinuation. **Conclusions:** This study provides novel insights into breastfeeding practices and maternal child health characteristics among mothers and infants affected by CHD in Saudi Arabia. The current study findings revealed that maternal CHD was significantly associated with advanced maternal age, family history, low birth weight, and coexisting chronic conditions, highlighting important genetic and perinatal risk factors. Despite these health burdens, breastfeeding initiation and duration did not significantly differ between CHD and non-CHD mothers, suggesting that maternal CHD alone does not hinder breastfeeding success.

## 1. Introduction

Breastfeeding is recognized as the optimal form of infant nutrition, providing essential nutrients, immunological protection, and psychological bonding benefits for both mother and child. The World Health Organization (WHO) and American Academy of Pediatrics recommend exclusive breastfeeding for the first six months of life and continued breastfeeding up to two years or beyond alongside complementary foods [1]. However, despite its well-documented benefits, breastfeeding rates (Global Breastfeeding Scorecard by UNICEF/WHO: ~48% of infants under 6 months old are exclusively breastfed) [2] remain suboptimal worldwide, with numerous maternal, neonatal, and systemic obstacles contributing to early cessation. Children with congenital heart disease (CHD) represent a unique group in which breastfeeding is especially critical. Breast milk provides immune protection, reduces infection risk, and may improve surgical outcomes and neurodevelopmental status in infants with CHD [3,4,5]. However, infants with CHD often experience feeding difficulties, poor weight gain, or require prolonged hospitalization, which complicates the establishment and continuation of breastfeeding [4,6]. Breastfeeding has been shown to confer multiple advantages in this vulnerable group. Studies report that breastfed infants with CHD demonstrate improved gastrointestinal tolerance, lower rates of infection, and better neurodevelopmental outcomes than those who are formula-fed [3,7,8]. Despite the well-established benefits of breastfeeding, infants with congenital heart disease (CHD) and their mothers face multiple barriers that contribute to early breastfeeding cessation. Infant-related barriers include prolonged hospitalizations, dependence on tube feeding, and delayed oral feeding readiness, all of which reduce opportunities for direct breastfeeding [9,10]. Mothers of infants with CHD frequently experience heightened psychological distress, fear about their infant’s condition, and uncertainty regarding feeding safety, which further discourage sustained breastfeeding [11]. Healthcare-system barriers particularly the limited availability of specialized lactation support within cardiac intensive care units and inconsistent or conflicting guidance from healthcare providers also hinder breastfeeding continuation. In addition, maternal CHD presents its own challenges, as women with CHD are at higher risk of pregnancy complications and postpartum fatigue or health instability, reducing their capacity to initiate or maintain breastfeeding [12]. Concerns regarding the safety of cardiac medications during lactation often lead mothers or clinicians to discontinue breastfeeding earlier than recommended, despite limited evidence of harm for many medications [13,14]. Furthermore, maternal fatigue, the burden of frequent medical appointments, and reduced physical endurance are additional obstacles that hinder sustained breastfeeding practices among this population.

In Saudi Arabia and the Middle East, breastfeeding initiation rates are generally high, yet the prevalence of exclusive breastfeeding at six months remains relatively low, ranging between 18% and 35% [15,16]. A study in Saudi Arabia found that while ~37.5% of mothers were exclusively breastfeeding at 2 weeks postpartum, this dropped to 19% by 2 months [17]. Another study reported only 16.3% of mothers exclusively breastfed [18]. Cultural practices, early introduction of formula, return to work, and insufficient lactation support have been consistently identified as barriers to exclusive breastfeeding in the region [19]. Studies examining breastfeeding among children with chronic illnesses in Saudi Arabia are scarce, but the available evidence indicates that mothers of hospitalized infants are less likely to sustain breastfeeding compared to mothers of healthy infants [20]. Although international studies have reported on breastfeeding challenges among infants with CHD and, separately, among mothers with chronic illnesses, there is a lack of research focusing on the dual burden of CHD in both mother and child. In Saudi Arabia, while general breastfeeding practices have been described, very limited data exist on high-risk populations such as CHD patients. Understanding the unique obstacles in this group will help in tailoring interventions, improving maternal and infant health, and aligning with Saudi Vision 2030 goals of enhancing maternal–child health. The aim of this study was to determine breastfeeding prevalence and to identify common obstacles among mothers with and without CHD whose children were diagnosed with CHD.

## 2. Methods

### 2.1. Study Design

This study employed a cross-sectional survey design to assess the prevalence of breastfeeding and the associated obstacles among mothers diagnosed with CHD whose children were also diagnosed with CHD. The study further evaluated breastfeeding practices, key barriers, and maternal health-related factors influencing breastfeeding outcomes in this high-risk population. Data were collected over an eight-month period, from 6 January 2025 to 17 August 2025, across multiple healthcare facilities in Saudi Arabia. Participating hospitals included tertiary care centers, pediatric cardiology units, and maternal child health clinics, where both mothers and infants with CHD are routinely managed. Mothers themselves were not required to have CHD; data were collected from both mothers with a confirmed diagnosis of CHD (childhood or adulthood) and those without CHD. Eligible participants were identified from cardiology outpatient clinics, pediatric cardiac wards, and maternal health units. Medical records were reviewed to confirm the child’s CHD diagnosis, and any maternal chronic diseases were identified through medical record review and a self-report questionnaire item asking whether the mother had ever been diagnosed with a chronic illness. For this study, chronic diseases included cardiovascular conditions (including maternal CHD), diabetes mellitus, hypertension, thyroid disorders, asthma, chronic renal disease, and autoimmune disorders such as lupus or rheumatoid arthritis. The inclusion criteria consisted of biological mothers who had attempted breastfeeding at any duration, mothers of children with a confirmed diagnosis of CHD based on medical records, and mothers who were willing and able to provide informed consent. Adoptive mothers were excluded, and although CHD diagnosis was confirmed, severity classification was not used as part of the inclusion criteria. The exclusion criteria included children without a confirmed CHD diagnosis, mothers with severe cognitive or psychiatric illness, and families unwilling to participate.

### 2.2. Data Collection Tool

A self-administered structured questionnaire was developed based on the prior literature and carefully reviewed by the research team for relevance, clarity, and methodological appropriateness. An initial set of 28 questions was finalized and submitted, along with the full proposal, to the Institutional Ethical Committee. The committee requested revisions, which the team addressed before resubmission. After two rounds of review and consultation with both the ethical committee and an expert panel, further refinements were made. The final approved questionnaire consisted of 22 items to enhance focus and feasibility. Following the committee’s recommendations, a pilot study involving 21 participants was conducted in December 2024 across two hospitals. Feedback from the pilot supported the tool’s feasibility, and the questionnaire demonstrated strong internal consistency, with a Cronbach’s alpha of 0.816. If any information was missing during data collection, those cases were not included in the study, and the incomplete records were removed from the dataset.

### 2.3. Instrument and Data Setting

A bilingual (Arabic–English) structured questionnaire was developed to ensure clarity and accessibility for all participants. It contained three sections: the first captured demographic and clinical details of mothers and their children, including maternal age, parity, education, CHD history, chronic diseases, pregnancy factors, and the child’s gender, gestational age, and birth weight. The second section focused on breastfeeding practices, including initiation and duration of exclusive breastfeeding. The third section examined obstacles to breastfeeding, such as maternal health issues, medication concerns, infant hospitalization, emotional stress, fatigue, and limited lactation support. The questionnaire required approximately 15–20 min to complete and was provided in both paper and electronic formats during hospital visits or via secure online platforms.

### 2.4. Ethics Approval and Consent to Participate

This study was approved by the Institutional Review Board (IRB) (# E-24-8489) at King Saud University (KSU) in Riyadh, Saudi Arabia. Before participating, all participants signed a consent form. All participants provided informed consent prior to data collection. Participation was voluntary. The methods used were in accordance with all relevant guidelines and regulations.

### 2.5. Sample Size

Based on the ethical committee’s discussion regarding sample size, it was suggested, drawing on findings from previously published studies, that a minimum of 300 participants would be required to achieve statistically significant results in line with the existing literature.

### 2.6. Statistical Analysis

We employed Microsoft Excel as our data input and analysis tool for comprehensive data management. For robust statistical analysis, we utilized SPSS version 24.0. In addition to calculating prevalence, we computed 95% confidence intervals to enhance statistical validity. To elucidate the associations between specific outcomes and the variables under consideration, we harnessed Pearson’s chi-square test and odds ratios (OR) to quantify risk factors and awareness associations. Throughout this study, we maintained a rigorous significance level of *p* < 0.05 to ensure our findings.

## 3. Results

A total of 419 mothers of children with congenital heart disease (CHD) participated in the study. Most mothers were aged 36–40 years (28.9%) or above 40 years (31.7%), while fewer were in the 23–30 age group (19.3%). Over half of the children were male (53.0%), and 47.0% were female. Regarding parity, 36.8% of mothers had 3–4 children, and 31.0% had 1–2 children. Almost all mothers were married (97.1%). Maternal health characteristics showed that 15.8% had CHD and 11.7% reported a family history of CHD, while 19.8% had other chronic illnesses. More than half of the infants were born at term (58.5%), whereas 41.5% were preterm. Low birth weight was observed in 56.1% of infants. The most common CHD diagnosis was atrial septal defect (46.5%), followed by ventricular septal defect (24.8%) and patent ductus arteriosus (19.3%), with less frequent conditions such as mitral stenosis, pulmonary stenosis, tricuspid regurgitation, and aortic stenosis making up the remainder (Table 1).

Table 2 shows the association between maternal age at delivery and various demographic and clinical factors. No significant differences were found across age groups for child’s gender, birth weight, or maternal chronic diseases. Significant associations did emerge for maternal CHD diagnosis (*p* = 0.004), with higher rates among mothers aged 31–35 years and those above 40. Family history of CHD also differed significantly (*p* < 0.001), being highest in the youngest age group and declining with age. Gestational age at delivery varied across maternal age groups (*p* < 0.001), with mothers aged 31–35 more likely to deliver at term, while preterm births were more common in the youngest mothers and those aged 36–40. The type of CHD in children also varied significantly with maternal age (*p* < 0.001): ASD was most frequent across all age groups, especially among mothers aged 23–30 and 36–40, VSD was more common in children of mothers ≥ 40, and mitral stenosis appeared exclusively in this oldest group. These findings suggest that maternal age is linked to both maternal health patterns and specific CHD types in offspring.

Table 3 presents the logistic regression analysis, which demonstrated several significant associations between maternal CHD diagnosis and demographic and clinical factors. Maternal age had a strong association, with women aged 31–35 years (OR = 4.75, 95% CI: 1.68–13.3, *p* = 0.0032) and ≥40 years (OR = 3.87, 95% CI: 1.42–10.51, *p* = 0.0079) showing significantly higher odds of CHD compared to those aged 23–30 years. Female children were more common among CHD mothers, with nearly twice the odds compared to male children (OR = 1.92, 95% CI: 1.1–3.28, *p* = 0.017). A positive family history of CHD was a strong predictor, increasing the odds more than four-fold (OR = 4.35, 95% CI: 2.26–8.34, *p* < 0.0001). Low birth weight was significantly associated with maternal CHD, with affected mothers being more than twice as likely to deliver infants with low birth weight (OR = 2.58, 95% CI: 1.43–4.66, *p* = 0.0016). Additionally, mothers with CHD were almost three times more likely to report having another chronic disease (OR = 2.84, 95% CI: 1.60–5.05, *p* = 0.0004). Child cardiac diagnoses also showed notable differences. Ventricular septal defect (VSD) was strikingly higher among children of CHD mothers (OR = 16.9, 95% CI: 5.00–57.2, *p* < 0.0001), while mitral stenosis also showed a strong association (OR = 11.8, 95% CI: 2.47–56.2, *p* = 0.0002). In contrast, atrial septal defect (ASD) was more common among non-CHD mothers, though this was not statistically significant (*p* = 0.155).

As seen in Table 4, logistic regression analysis showed no significant association between maternal CHD status and breastfeeding practices. Most mothers initiated breastfeeding, with similar rates among CHD (92.4%) and non-CHD (90.4%) mothers (OR = 1.25, 95% CI: 0.47–3.30, *p* = 0.64). Furthermore, breastfeeding the child was reported by 62.1% of CHD mothers and 59.2% of non-CHD mothers, with no significant difference (OR = 1.10, *p* = 0.705). Duration of breastfeeding also showed no meaningful association; although CHD mothers reported slightly higher rates of breastfeeding for 12–24 months, this was not statistically significant. Moreover, discussions about breastfeeding’s benefits and support from healthcare professionals were comparable across groups, with no significant effects observed. Overall, maternal CHD status did not significantly influence breastfeeding initiation, duration, or professional support.

Table 5 summarizes the association between obstacles to breastfeeding and maternal congenital heart disease (CHD). Mothers with CHD were significantly less likely to report facing breastfeeding obstacles compared to those without CHD (27.3% vs. 43.1%, OR = 0.54, 95% CI: 0.30–0.97, *p* = 0.04). Receiving assistance in overcoming these obstacles did not differ significantly between CHD and non-CHD mothers (33.3% vs. 39.1%, OR = 0.80, 95% CI: 0.46–1.4, *p* = 0.44). When examining specific reasons for discontinuing or not initiating breastfeeding, notable differences emerged. None of the CHD mothers reported pain as a reason for stopping breastfeeding, whereas 21.2% of non-CHD mothers did (OR = 1.23, 95% CI: 0.86–1.76, *p* = 0.24). Similarly, no CHD mothers stated they did not want to breastfeed, while 12.1% of non-CHD mothers reported this reason (*p* = 0.05). In addition, fewer CHD mothers perceived breastfeeding as too tiring compared with non-CHD mothers (12.1% vs. 24.9%, *p* = 0.05).

## 4. Discussion

In this study involving 419 mothers of children with CHD, several maternal, neonatal, and lesion-type patterns were identified that partially align with global evidence while also highlighting characteristics unique to our cohort. A notable proportion of mothers were of advanced maternal age, with 28.9% aged 36–40 years and 31.7% aged ≥ 40 years. These proportions appear higher than national maternal age distributions reported in Saudi Arabia, suggesting that older maternal age may be more prominent within this CHD-affected cohort [21]. Advanced maternal age has been increasingly recognized as a risk factor for congenital heart defects [22,23]. The presence of maternal CHD in 15.8% and a family history of CHD in 11.7% underscores the likelihood of heritable or familial predisposition, consistent with studies showing increased recurrence risk among affected mothers [24,25,26]. The current study demonstrated substantial perinatal adversity within this cohort, with 41.5% of children born preterm and 56.1% classified as having low birth weight. These findings are in line with prior research indicating that fetuses with CHD often experience intrauterine growth restriction and preterm delivery due to impaired hemodynamics and placental insufficiency [27]. The dominance of atrial septal defect (46.5%), followed by VSD (24.8%) and PDA (19.3%), mirrors global CHD epidemiology trends where septal defects remain among the most common lesions [28]. These patterns have several implications. The concentration of older mothers and familial CHD suggests that screening and genetic counseling might be prioritized in these populations. The high rates of preterm birth and low birth weight reinforce the need for enhanced perinatal care and nutritional support in CHD-affected pregnancies.

The current study reported that mothers aged 31–35 years and those aged ≥40 years had a significantly higher prevalence of CHD diagnosis compared to younger mothers (*p* = 0.004). This finding is consistent with the concept that maternal aging contributes to increased cardiac risk or vulnerability, possibly through age-related vascular, metabolic, or epigenetic changes [29,30]. Some epidemiologic studies have reported higher CHD risk in offspring born to older mothers, especially for septal defects such as ventricular septal defect (VSD) [31,32,33]. At the same time, meta-analyses are mixed; one large review found no consistent significant association between maternal age and CHD risk overall, though a modest risk increase emerges after adjustment for confounds [34]. This suggests that maternal age may act in concert with genetic predispositions and environmental modifiers, rather than as an independent driver, as supported by cohort studies demonstrating increased risk of congenital heart defects with advanced maternal age in combination with familial or environmental factors [29]. In this study, we also found that the proportion of mothers with a family history of CHD declined with increasing maternal age (*p* < 0.001). This pattern may reflect survivorship or generational shifts in CHD diagnosis, though the precise mechanism is unclear. A family history remains a well-recognized risk factor for CHD recurrence [26]. In the current study on pregnancy outcomes, our data also revealed significant variation in gestational age by maternal age (*p* < 0.001). Mothers aged 31–35 years had the highest term delivery rates, whereas preterm birth was more common in very young and middle-aged mothers. This aligns with studies in the literature showing that extremes of maternal age (both younger and older) are associated with preterm delivery risks [35]. Furthermore, the distribution of CHD subtypes varied by maternal age (*p* < 0.001): atrial septal defect (ASD) was dominant across most age groups, but VSD prevalence was particularly elevated in children of mothers ≥ 40 years; mitral stenosis appeared exclusively in this oldest group. Such differences may point toward age-related susceptibility to particular cardiac morphogenesis pathways or differential detection biases.

In this current study, logistic regression showed several notable associations between maternal factors and the presence of maternal congenital heart disease (CHD). Women aged 31–35 years had 4.75-fold greater odds (95% CI: 1.68–13.3, *p* = 0.0032), and women aged ≥ 40 years had 3.87-fold greater odds (95% CI: 1.42–10.51, *p* = 0.0079) of CHD compared to those aged 23–30 years. These findings suggest a U-shaped age profile in maternal CHD risk, where middle and advanced maternal age may reflect cumulative exposures or underlying cardiovascular vulnerability. This finding align with previous publish study, increasing maternal age could expose underlying cardiovascular risks, as age-associated alterations accumulated over time may impair vascular reactivity [36].

The association between female children and maternal CHD (OR = 1.92, 95% CI: 1.10–3.28, *p* = 0.017) raises intriguing questions about sex-specific vulnerability or ascertainment bias in families where mothers have CHD. Female infant sex has been associated with maternal CHD in some studies, possibly due to sex-specific differences in fetal susceptibility to maternal cardiovascular and hemodynamic conditions during pregnancy. Hormonal and genetic factors may contribute to this differential risk between male and female fetuses [34,37]. A robust positive family history was also a strong predictor: mothers reporting CHD in first-degree relatives had over four times higher odds of CHD themselves (OR = 4.35, 95% CI: 2.26–8.34, *p* < 0.0001). This aligns with classic recurrence risk models showing higher transmission risk when the parent is affected [38,39]. Moreover, in this study, low birth weight status of the child was significantly associated with maternal CHD (OR = 2.58, 95% CI: 1.43–4.66, *p* = 0.0016). This may reflect fetomaternal hemodynamic stress, placental insufficiency, or shared vascular pathology impacting intrauterine growth [40]. Maternal comorbid chronic disease also correlated with higher odds of CHD (OR = 2.84, 95% CI: 1.60–5.05, *p* = 0.0004), suggesting that systemic maternal disease burden might contribute to cardiac vulnerability or shared developmental pathways. This finding aligns with previous published studies, suggesting that increasing maternal age may expose underlying cardiovascular risks, as age-associated alterations accumulated over time can impair vascular reactivity [41]. In this study, we found that the most striking associations emerged in child CHD types. Children of CHD mothers showed dramatically elevated odds of ventricular septal defect (VSD) (OR = 16.9, 95% CI: 5.00–57.2, *p* < 0.0001) and mitral stenosis (OR = 11.8, 95% CI: 2.47–56.2, *p* = 0.0002). By contrast, atrial septal defect (ASD) was more frequent in non-CHD mothers (though not statistically significant, *p* = 0.155). The strong maternal offspring linkage in VSD is biologically plausible given that VSD is one of the most common CHD subtypes and is known to exhibit familial clustering [42,43,44]. Indeed, experimental models also suggest that maternal factors (e.g., age or epigenetic modifiers) can modulate penetrance of VSD in genetically predisposed embryos [45].

In our study, logistic regression analysis showed no significant differences in breastfeeding initiation, duration, or professional support between mothers with CHD and those without. Initiation rates were similar (92.4% vs. 90.4%), and the proportion breastfeeding their CHD-diagnosed child was comparable (62.1% vs. 59.2%). Duration of breastfeeding and discussions with healthcare professionals also did not differ significantly, despite the physiological and logistical challenges associated with CHD [46,47]. Similar to findings from a Swedish registry, most women with CHD in our study also initiated breastfeeding, although at slightly lower rates compared to mothers without CHD [46]. Holstad et al.’s analysis also revealed that the presence of CHD itself was associated with non-breastfeeding in unadjusted models, but many heart-related factors became nonsignificant in adjusted analyses [46].

Other studies similarly show that although CHD increases risks, many affected mothers can breastfeed successfully; in one recent study of first-time mothers with CHD, early breastfeeding rates were only slightly lower, and heart-related factors no longer predicted breastfeeding outcomes by four weeks postpartum [46]. Moreover, clinical guidance often supports breastfeeding in mothers with CHD unless contraindications exist [48]. Our findings showed that mothers with CHD were less likely to report breastfeeding obstacles than those without CHD, but both groups received similar levels of assistance to manage these challenges. This suggests that CHD status did not intensify breastfeeding barriers, possibly due to greater motivation, increased clinical attention, or shared challenges across both groups [49,50].

When examining specific reasons for breastfeeding discontinuation or non-initiation, we found that none of the mothers with CHD reported pain as a barrier, whereas 21.2% of mothers without CHD did, although this difference was not statistically significant (*p* = 0.24). Similarly, no CHD mothers reported unwillingness to breastfeed, compared with 12.1% of non-CHD mothers (*p* = 0.05), and fewer CHD mothers perceived breastfeeding as “too tiring” (12.1% vs. 24.9%, *p* = 0.05). These patterns may reflect differences in perception or selection characteristics, as mothers with CHD who deliver and participate in research may represent a healthier or more medically engaged subgroup. Rather than attributing these findings to maternal “motivation,” it is also possible that CHD mothers receive more specialized follow-up or structured counseling as part of high-risk care pathways, which could reduce perceived obstacles. This aligns with the finding that CHD mothers did not report pain, reluctance, or fatigue as strongly; perhaps CHD mothers fall more into clusters of physiological challenges rather than maternal reluctance or perception-based obstacles [49]. A scoping review of 14 studies on infants with CHD revealed that although many mothers of infants with CHD can produce sufficient milk volumes by one-month postpartum, direct breastfeeding rates remain low due to inconsistent support and messaging from healthcare providers [51]. Consistently, our results showed no significant difference between CHD and non-CHD mothers regarding receipt of assistance to overcome breastfeeding difficulties, underscoring the importance of individualized care and proactive lactation support rather than assumptions about CHD as an inherent barrier. This study findings suggest that maternal CHD should not automatically be viewed as a barrier to breastfeeding; rather, individualized care, anticipatory support, and attention to common breastfeeding challenges remain critical.

## 5. Limitations

In this study, there were a few limitations that should be acknowledged. First, the cross-sectional design limits causal inference between maternal characteristics, CHD status, and breastfeeding outcomes, and although the sample size of 419 mothers provided sufficient power for primary analyses, it limited subgroup comparisons, particularly across different CHD lesion types. Second, CHD severity (e.g., mild, moderate, or complex) was not classified in the medical records used for this study, which may influence breastfeeding capacity and clinical needs. Third, the cross-sectional design restricts causal interpretation between maternal characteristics, CHD status, and breastfeeding outcomes. Fourth, self-reported breastfeeding practices and perceived obstacles are vulnerable to recall bias and social desirability effects. Fifth, recruitment from tertiary hospitals may introduce selection bias by overrepresenting more complex CHD cases and mothers with greater health-seeking behavior, thereby reducing generalizability to community populations. Finally, although some demographic factors were collected, broader socioeconomic variables were not assessed, which may also influence breastfeeding behaviors. Despite these limitations, the study contributes important regional evidence supporting future policy and research on CHD and breastfeeding.

## 6. Conclusions

The current study findings revealed that maternal CHD was significantly associated with advanced maternal age, family history, low birth weight, and coexisting chronic conditions, highlighting important genetic and perinatal risk factors. Despite these health burdens, breastfeeding initiation and duration did not significantly differ between CHD and non-CHD mothers, suggesting that maternal CHD alone does not hinder breastfeeding success. Interestingly, CHD mothers reported fewer perceived breastfeeding obstacles, potentially reflecting greater motivation, increased medical attention, or selective participation among healthier women with CHD. These findings underscore the importance of early risk assessment, genetic counseling, and enhanced perinatal and lactation support for mothers and infants with CHD. Targeted interventions focusing on both medical and psychosocial needs could improve breastfeeding continuity and overall maternal child health outcomes, aligning with national goals to promote optimal infant nutrition and maternal well-being. Integrating lactation consultants into routine CHD care pathways may strengthen breastfeeding support for both mothers with CHD and mothers of infants with CHD. Future research should adopt prospective cohort or interventional designs to better capture causal relationships and evaluate the impact of targeted breastfeeding support programs in CHD populations. Studies with larger, multicenter samples and detailed CHD severity classifications are needed to improve generalizability and refine clinical recommendations.

## Figures and Tables

**Table 1:** Demographic information on mothers and children.

Factor	Categories	n (%)
Mother’s age time of delivery	23–30	81 (19.3)
	31–35	84 (20.0)
	36–40	121 (28.9)
	40 or more	133 (31.7)
Child’s gender	Male	222 (53.0)
	Female	197 (47.0)
Number of children	1 to 2	130 (31.0)
	3 to 4	154 (36.8)
	4 to 6	100 (23.9)
	7 or more	35 (8.4)
Marital status	Married	407 (97.1)
	Divorced	12 (2.9)
Economic status	0 to 5000	108 (25.8)
	5001 to 10,000	179 (42.7)
	10,000 to 20,000	107 (25.5)
	More than 20,000	25 (6.0)
Mothers with diagnosed CHD	Yes	66 (15.8)
	No	353 (84.2)
Mother with family history of CHD	Yes	49 (11.7)
	No	370 (88.3)
In which week of your pregnancy was your child delivered?	At term	245 (58.5)
	Preterm	174 (41.5)
What was your child’s birth weight in kg?	Adequate weight	184 (43.9)
	Low birth weight	235 (56.1)
Type of CHD in child	Aortic stenosis	6 (1.4)
	Atrial septal defect (ASD)	195 (46.5)
	Mitral stenosis	16 (3.8)
	Patent ductus arteriosus (PDA)	81 (19.3)
	Pulmonary stenosis	9 (2.1)
	Tricuspid regurgitation	8 (1.9)
	Ventricular septal defect (VSD)	104 (24.8)
Have you ever been diagnosed with any chronic disease?	Yes	83 (19.8)
	No	336 (80.2)

**Table 2:** Association of demographic information with maternal age.

		Age				
Factors	Categories	23–30	31–35	36–40	40–More	Chi-Square (*p*-Value)
Child’s gender	Male	40 (49.4)	38 (45.2)	67 (55.4)	77 (57.9)	4.09 (0.26)
	Female	41 (50.6)	46 (54.8)	54 (44.6)	56 (42.1)	
Were you diagnosed with CHD?	Yes	5 (6.2)	20 (23.8)	14 (11.6)	27 (20.3)	13.37 (0.004)
	No	76 (93.8)	64 (76.2)	107 (88.4)	106 (79.7)	
Mother with family history of CHD	Yes	16 (19.8)	14 (16.7)	16 (13.2)	3 (2.3)	18.8 (0.000)
	No	65 (80.2)	70 (83.3)	105 (86.8)	130 (97.7)	
In which week of your pregnancy was your child delivered?	At term	38 (46.9)	60 (71.4)	57 (47.1)	90 (67.7)	21.34 (0.000)
	Preterm	43 (53.1)	24 (28.6)	64 (52.9)	43 (32.3)	
What was your child’s birth weight in kg?	Adequate weight	32 (39.5)	33 (39.3)	63 (52.1)	56 (42.1)	4.81 (0.186)
	Low birth weight	49 (60.5)	51 (60.7)	58 (47.9)	77 (57.9)	
Have you ever been diagnosed with any chronic disease?	Yes	17 (21.0)	15 (17.9)	22 (18.2)	29 (21.8)	0.807 (0.84)
	No	64 (79.0)	69 (82.1)	99 (81.8)	104 (78.2)	
Child’s diagnosis: type of CHD	Aortic stenosis	0 (0.0)	3 (3.6)	3 (2.5)	0 (0.0)	69.71 (0.000)
	Atrial septal defect (ASD)	43 (53.1)	31 (36.9)	65 (53.7)	56 (42.1)	
	Mitral stenosis	0 (0.0)	0 (0.0)	0 (0.0)	16 (12.0)	
	Patent ductus arteriosus (PDA)	21 (25.9)	27 (32.1)	18 (14.9)	15 (11.3)	
	Pulmonary stenosis	0 (0.0)	3 (3.6)	3 (2.5)	3 (2.3)	
	Tricuspid regurgitation	0 (0.0)	0 (0.0)	3 (2.5)	5 (3.8)	
	Ventricular septal defect (VSD)	17 (21.0)	20 (23.8)	29 (24.0)	38 (28.6)	

**Table 3:** Association between demographic factors and mothers diagnosed with congenital heart disease (CHD).

Factor	Categories	Yes (66)	95% CI	*p* = Value	No (353)
Mother’s age	23–30	5 (7.6)	Ref-1		76 (21.5)
	31–35	20 (30.3)	4.75 (1.68–13.3)	0.0032	64 (18.1)
	36–40	14 (21.2)	1.98 (0.68–5.75)	0.2	107 (30.3)
	40–more	27 (40.9)	3.87 (1.42–10.51)	0.0079	106 (30.0)
Child’s gender	Male	26 (39.4)	0.52 (0.30–0.89)	0.017	196 (55.5)
	Female	40 (60.6)	1.92 (1.1–3.28)	0.017	157 (44.5)
Mother with family history of CHD	Yes	19 (28.8)	4.35 (2.26–8.34)	<0.0001	30 (8.5)
	No	47 (71.2)	Ref-1		323 (91.5)
In which week of your pregnancy was your child delivered?	At term	39 (59.1)	1.03 (0.60–1.75)	0.91	206 (58.4)
	Preterm	27 (40.9)	0.97 (0.56–1.65)	0.91	147 (41.6)
What was your child’s birth weight in kg?	Adequate weight	17 (25.8)	Ref-1		167 (47.3)
	Low birth weight	49 (74.2)	2.58 (1.43–4.66)	0.0016	186 (52.7)
Have you ever been diagnosed with any chronic disease?	Yes	24 (36.4)	2.84 (1.60–5.05)	0.0004	59 (16.7)
	No	42 (63.6)	Ref-1		294 (83.3)
Child diagnosis: type of CHD	Aortic stenosis	0 (0.0)			6 (1.7)
	Atrial septal defect (ASD)	17 (25.8)	2.48 (0.70–8.71)	0.155	178 (50.4)
	Mitral stenosis	5 (7.6)	11.8 (2.47–56.20	0.0002	11 (3.1)
	Patent ductus arteriosus (PDA)	3 (4.5)	Ref-1		78 (22.1)
	Pulmonary stenosis	0 (0.0)			9 (2.5)
	Tricuspid regurgitation	0 (0.0)			8 (2.3)
	Ventricular septal defect (VSD)	41 (62.1)	16.9 (5.00–57.2)	<0.0001	63 (17.8)

**Table 4:** Association between breastfeeding practices and mothers diagnosed with congenital heart disease (CHD).

			Mother Diagnosed with Congenital Heart Disease (CHD)					
Factor	Categories	n (%)	Yes (66)	95% CI	*p*-Value	No (353)	95% CI	*p*-Value
Did you initiate breastfeeding?	Yes	380 (90.7)	61 (92.4)	1.25 (0.47–3.30)	0.64	319 (90.4)	0.96 (0.59–1.56)	0.87
	No	39 (9.3)	5 (7.6)	Ref.		34 (9.6)	Ref.	
Did you breastfeed your child who was diagnosed with CHD?	Yes	250 (59.7)	41 (62.1)	1.10 (0.64–1.89)	0.705	209 (59.2)	0.98 (0.73–1.30)	0.89
	No	169 (40.3)	25 (37.9)	Ref.		144 (40.8)	Ref.	
How long was your child breastfed for?	Less than 6 months	280 (66.8)	40 (60.6)	0.53 (0.16–1.69)	0.28	240 (68.0)	1.16 (0.52–2.59)	0.701
	Between 6 months and 12 months	58 (13.8)	4 (6.1)	0.25 (0.05–1.15)	0.076	54 (15.3)	1.26 (0.53–3.0)	0.58
	Between 12 months and 24 months	66 (15.8)	18 (27.3)	1.02 (0.30–3.46)	0.97	48 (13.6)	0.99 (0.41–2.34)	0.98
	More than 24 months	15 (3.6)	4 (6.1)	Ref.		11 (3.1)	Ref.	
Did your healthcare provider discuss the benefits of breastfeeding?	Yes	269 (64.2)	40 (60.6)	0.85 (0.50–1.46)	0.57	229 (64.9)	1.02 (0.76–1.38)	0.84
	No	150 (35.8)	26 (39.4)	Ref.		124 (35.1)	Ref.	
Did you receive any support or guidance from healthcare professionals?	Yes	294 (70.2)	45 (68.2)	0.91 (0.52–1.59)	0.74	249 (70.5)	1.01 (0.74–1.38)	0.91
	No	125 (29.8)	21 (31.8)			104 (29.5)		

**Table 5:** Association between obstacles to breastfeeding and mothers diagnosed with congenital heart disease (CHD).

			Mother Diagnosed with Congenital Heart Disease (CHD)					
Factor	Categories	n (%)	Yes (66)	95% CI	*p*-Value	No (353)	95% CI	*p*-Value
Did you face any obstacles while breastfeeding?	Yes	170 (40.6)	18 (27.3)	0.54 (0.30–0.97)	0.04	152 (43.1)	1.1 (0.83–1.47)	0.48
	No	249 (59.4)	48 (72.7)	Ref.		201 (56.9)	Ref.	
Did you receive any assistance in overcoming these obstacles?	Yes	160 (38.2)	22 (33.3)	0.80 (0.46–1.4)	0.44	138 (39.1)	1.03 (0.77–1.38)	0.79
	No	259 (61.8)	44 (66.7)	Ref.		215 (60.9)	Ref.	
What were the problems you faced that led you to stop/not start breastfeeding your child? Mother’s health problems	Yes	100 (23.9)	16 (24.2)	1.02 (0.55–1.87)	0.94	84 (23.8)	0.99 (0.71–1.38)	0.98
	No	319 (76.1)	50 (75.8)	Ref.		269 (76.2)	Ref.	
What were the problems you faced that led you to stop/not start breastfeeding your child? New pregnancy or OCP	Yes	32 (7.6)	8 (12.1)	1.02 (0.55–1.87)	0.22	24 (6.8)	0.88 (0.50–1.52)	0.65
	No	387 (92.4)	58 (87.9)	Ref.		329 (93.2)	Ref.	
What were the problems you faced that led you to stop/not start breastfeeding your child? Mother did not want to breastfeed	Yes	44 (10.5)	0 (0.0)	0.063 (0.003–1.04)	0.05	44 (12.5)	1.21 (0.77–1.89)	0.39
	No	375 (89.5)	66 (100)	Ref.		309 (87.5)	Ref.	
What were the problems you faced that led you to stop/not start breastfeeding your child? Mother returned to work	Yes	54 (12.9)	14 (21.2)	1.81 (0.94–3.50)	0.07	40 (11.3)	0.86 (0.55–1.33)	0.51
	No	365 (87.1)	52 (78.8)	Ref.		313 (88.7)	Ref.	
What were the problems you faced that led you to stop/not start breastfeeding your child? Pain	Yes	75 (17.9)	0 (0.0)	0.034 (0.002–0.56)	0.018	75 (21.2)	1.23 (0.86–1.76)	0.24
	No	344 (82.1)	66 (100)	Ref.		278 (78.8)	Ref.	
What were the problems you faced that led you to stop/not start breastfeeding your child? Too tiring	Yes	96 (22.9)	8 (12.1)	0.46 (0.21–1.0)	0.05	88 (24.9)	1.11 (0.80–1.55)	0.51
	No	323 (77.1)	58 (87.9)	Ref.		265 (75.1)	Ref.	
What were the problems you faced that led you to stop/not start breastfeeding your child? There is not enough time	Yes	62 (14.8)	11 (16.7)	1.15 (0.57–2.3)	0.69	51 (14.4)	0.97 (0.65–1.45)	0.89
	No	357 (85.2)	55 (83.3)	Ref.		302 (85.6)	Ref.	
What were the problems you faced that led you to stop/not start breastfeeding your child? Embarrassment feeding in public	Yes	24 (5.7)	3 (4.5)	0.78 (0.22–2.67)	0.69	21 (5.9)	1.04 (0.56–1.90)	0.89
	No	395 (94.3)	63 (95.5)	Ref.		332 (94.1)	Ref.	
What were the problems you faced that led you to stop/not start breastfeeding your child? I have other children to take care of	Yes	78 (18.6)	9 (13.6)	0.69 (0.32–1.45)	0.32	69 (19.5)	1.06 (0.74–1.52)	0.74
	No	341 (81.4)	57 (86.4)	Ref.		284 (80.5)	Ref.	

## Data Availability

Some data are not publicly available due to ethical and privacy restrictions. Other datasets used and/or analyzed during this study are available from the corresponding author upon reasonable request.

## Abbreviations

CHD	Congenital heart disease
IRB	Institutional review board
ASD	Atrial septal defect
PDA	Patent ductus arteriosus
VSD	Ventricular septal defect
SPSS	Statistical Package for the Social Sciences
IBM	International Business Machines
